# Structural predictions of the functions of membrane proteins from HDX-MS

**DOI:** 10.1042/BST20190880

**Published:** 2020-06-29

**Authors:** Andy M. Lau, Ruyu Jia, Richard T. Bradshaw, Argyris Politis

**Affiliations:** Department of Chemistry, King's College London, London, U.K.

**Keywords:** hydrogen-deuterium exchange mass spectrometry, integrative modelling, membrane proteins, transport

## Abstract

HDX-MS has emerged as a powerful tool to interrogate the structure and dynamics of proteins and their complexes. Recent advances in the methodology and instrumentation have enabled the application of HDX-MS to membrane proteins. Such targets are challenging to investigate with conventional strategies. Developing new tools are therefore pertinent for improving our fundamental knowledge of how membrane proteins function in the cell. Importantly, investigating this central class of biomolecules within their native lipid environment remains a challenge but also a key goal ahead. In this short review, we outline recent progresses in dissecting the conformational mechanisms of membrane proteins using HDX-MS. We further describe how the use of computational strategies can aid the interpretation of experimental data and enable visualisation of otherwise intractable membrane protein states. This unique integration of experiments with computations holds significant potential for future applications.

## HDX-MS of membrane proteins

Membrane proteins play critical roles in higher organisms and are responsible for diverse cellular functions such as signalling and molecular transport. Understanding the mechanism underpinning membrane-related function requires detailed characterisation of membrane protein structure and dynamics. Impressive advances in high-resolution structural methods such as cryo-electron microscopy (EM) and X-ray crystallography, have contributed valuable snapshots of membrane proteins within lipid environments, including P-glycoprotein [1], betaine transporter [2] and GPCRs [3]. Other emerging structural techniques such as pulsed electron double resonance (PELDOR or DEER) have contributed invaluable insights into membrane protein structural dynamics [4–6]. While high resolution structures of membrane nanomachines are undeniably pertinent, our ability to leverage structure against function, requires us to have access to their conformational changes and functional states.

Structural MS has emerged as a powerful tool for characterising protein structure and function, complementing high-resolution methods [7–9]. Over the last decade, progresses in native MS have allowed the retention of intact membrane protein structures into the gas phase of a mass spectrometer and more spectacularly, to characterise lipid binding events to such proteins [9–16]. While powerful, native MS alone, offers little information on protein dynamics. HDX-MS, on the other hand, provides an extremely sensitive method for interrogating the conformational dynamics of proteins and their complexes via monitoring the exchange of hydrogens to deuterium [17–19]. The main advantage of HDX-MS over other biophysical techniques is that it is able to tolerate a wide range of complex environments (e.g. lipids), able to monitor changes in structure across populations of proteins [20–26], all while without requiring covalent modification. This is particularly important for membrane-embedded proteins since introducing modifications can greatly lower expression yield, introduce heterogeneity and affect protein dynamics [21,27].

The most functionally relevant information for membrane proteins arises from differential HDX (**Δ**HDX), which determines and localises regions of significant structural change between two states of a protein or complex (e.g. a protein alone compared with protein with ligand) [22,23,28]. This provides a valuable readout for monitoring the conformational responses of proteins upon stimulation, both temporally and spatially. To aid the visualisation and interpretation of HDX-MS data, a number of computational software packages including Deuteros [29], MemHDX [30], HDX-Viewer [31] have been developed and are readily available to the community.

## HDX-MS in lipid environments

Developments in HDX-MS methodologies have allowed the investigation of membrane protein dynamics under previously inaccessible conditions and environments. While detergent micelles remain the primary choice for membrane protein solubilisation, recent advances have enabled the interrogation of membrane protein dynamics in lipid-based environments, reminiscent of their natural conditions [22,26,32]. More specifically, researchers have used HDX-MS to probe the structural dynamics of membrane proteins embedded in SMALPs [26], nanodiscs [20,22] and proteoliposomes [21]. Although HDX is tolerant of lipids and other complex environments, interfacing lipids with the fast separation and digestions required for HDX-MS can lead to fouling of the liquid chromatography (LC) and digestion columns. These challenges can be mitigated through a number of workflow procedures including using a sawtooth LC method, running ‘clean blank’ injections, and pepsin washes after each lipid-containing sample to minimise the build-up of lipids [32 33]. Extensive details of how to manipulate the lipid environments surrounding membrane proteins for exploring lipid-mediated conformational changes can be found in a recently published protocol [32]. In this review, we instead focus on advances in interpreting and functionally characterising HDX-MS experiments, in particular via the combination of computational modelling with experimental observations.

## Structural predictions from HDX-MS

Restraint-based modelling using data derived from structural MS methods are becoming increasingly useful for assembling multi-subunit protein complexes and tracking large-scale conformational changes. Examples of such utility can be demonstrated across a number of MS hyphenated techniques including chemical cross-linking (XL)-MS [31,34–36], ion mobility (IM)-MS [37], and native MS [14,38]. There are currently, however, limited examples of how HDX-MS data can be used in a similar fashion. Below provides a brief summary of current efforts employed in the modelling of proteins using HDX-MS data.

**Figure 1.**
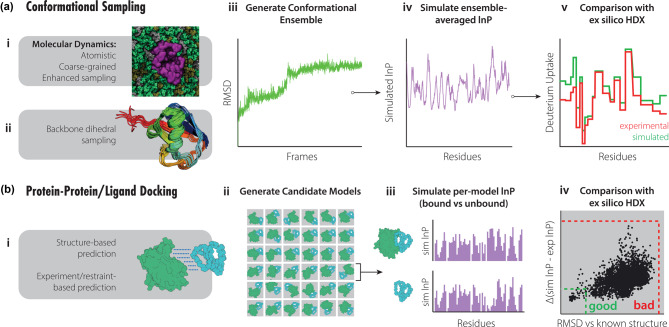
Methods of comparison between simulated and experimental HDX. (**a**) Protein structures representative of experimental HDX conditions can be leveraged against models generated from conformational sampling techniques. (i) Molecular dynamics simulations or (ii) dihedral space sampling can be used to generate diverse conformational ensembles (iii). (iv,v) Protection factors (typically used as the natural logarithm of the protection factor; lnP) can be simulated from models and averaged across trajectories to provide ensemble-averaged approximations that can be compared with experimental HDX values. (**b**) Docking methods relying on (i) structural complementarity or guided by experimental restraints can be used to generate libraries of candidate models (ii). (iii,iv) Simulated protection factors for bound vs unbound states can be compared with experimentally calculated values and used to score plausible models.

Structural modelling with HDX-MS typically envisions the leveraging of protein models derived from various computational methods, against experimental measurements (Figure 1). The objective of these approaches is to assess HDX-MS data collected from an experimental state of the protein (e.g. protein with ligand) against predicted data for each modelled conformation, and thereby identify or generate three-dimensional models that agree with the experimental observations. Integrating HDX-MS data directly into a computational modelling process can thereby provide a conduit between the structure of the protein target, its particular conformation within the experimental state, and the function of the protein. The accuracy of any integrative modelling method depends upon the suitability of sampling of protein conformations, and the accuracy of the equations and assumptions used to predict experimental observables from structural models. The suitability of different methods of model generation, HDX protection factor calculation, and rationale behind amide exchange have been evaluated by several key studies.

Recent work by Devaurs et al. [39] demonstrated how HDX-MS data can be used to select for accurate conformational representations of the immune complement protein C3d. In this study, Devaurs et al. systematically compare the suitability of models from various sources, in corroborating with experimental measurements. Models of C3d were obtained from either crystal structures, molecular dynamics simulations or generated from coarse-grained conformational sampling. The commonly used phenomenological approximation method [40,41] was used to simulate deuterium uptake metrics from these models and were compared with experimental values. The authors observed that the best agreement was obtained from models generated via coarse grained conformational sampling, highlighting that adequate conformational sampling is a prerequisite for agreement of predicted and observed deuterium exchange.

A study by the Gross lab [42] combined XL-MS and HDX-MS to generate a structure of Interleukin 7 in complex with its cognate α-receptor IL-7Rα. The authors showed that only a few cross-links are needed to generate high confidence structures, but note that this is largely dependent on system in question. The authors performed cross-link guided docking using each combination of cross-links to generate candidate models. Two areas were identified from differential HDX-MS to be protected upon binding of IL7 to receptor.

Multiple methods of deriving *in silico* protection factor (PF) or deuterium uptake exist. For each backbone amide, the PF quantifies the degree to which the observed deuterium exchange rate is reduced compared with the intrinsic (unprotected) amide exchange rate, as a result of the protein fold [43]. There are several methods of predicting PF, but all use the thermodynamic concept that PF represents the equilibrium constant for local protein unfolding that results in exposure of the amide to solvent and subsequent possibility for exchange. PF prediction methods can be broadly clustered into one of two categories. The first group are methods that use structure-based scoring functions as a proxy for the ΔG of local unfolding. For example, the widely used phenomenological approximation estimates PF as a function of the number of hydrogen bonds and the number of non-hydrogen atoms within a radial distance threshold of the amide hydrogen [40,41]. The second category of predictions directly derive equilibrium constants from a structural ensemble, for example by counting fractional populations of ‘closed’ and ‘open’ states for each amide position from MD simulations [44], or sampling of the unfolded and folded protein states by methods such as COREX [45].

One study by Mohammadiarani et al. [46], aimed to assess the accuracy of HDX-MS prediction models by comparing seven different models of calculating PF for the same protein datasets. Mohammadiarani et al. performed simulations of 3 homologous G-protein signalling proteins (RGS4, RGS8 and RGS19) and assessed the performance of each method in matching experimental measurements. The authors conclude that fractional-population models outperform commonly used empirical models such as phenomenological approximation. Of note, the authors describe that the inclusion of solvent accessible surface area (SASA) together with a distance measurement between amide hydrogens from the first polar atom in proteins, provides an accuracy boost to PF approximations.

In another study, McAllister and Konermann review to what extent experimentally observed HDX could be rationalised through hydrogen bonding or solvent accessibility [47]. The authors perform molecular dynamics simulations of ubiquitin, monitoring each amide position over the course of the simulation. Interestingly, McAllister and Konermann found that while most of the protons could be accounted for through either conformation-induced changes in hydrogen bonding or solvent accessibility, protons at 15 sites were unaccounted for. They further analysed the solvation properties of protected and unprotected surface amides and found no differences, suggesting that restricted waters are unlikely to cause anomalous protection of amides. The authors finish with the outlook that it may be time to pursue quantum mechanical calculations of proteins and look at protein–solvent interactions.

## MD and HDX-MS

An area of intense interest is the combined exploitation of HDX-MS and MD simulations to monitor the conformational dynamics of proteins and their complexes. The marriage of HDX-MS and MD has led to fruitful discoveries for soluble proteins, for example in the areas of cellular GTPases [48], aggregation of prion proteins [49] and how ions can modulate protein function [50]. HDX-MS and MD has also been applied to membrane proteins. Zhang et al. [51] utilised crystallography to resolve the inactive conformation of the glucagon receptor (GCGR; a GPCR). The authors perform MD simulations to gain a representative model of the apo-GCGR and perform HDX-MS to probe the dynamics of the receptor in complex with mAb1 and mAb23 antibodies, which act as antagonist and inverse agonists of the GCGR, respectively. Using differential HDX-MS, the authors were able to assign both the binding regions of mAb1 and mAb23 to GCGR, and also identify differences between the HDX-MS profiles of mAb1 and mAb23-bound complexes. The authors collate these findings to describe the structural basis for how binding of mAb1 and mAb23 to GCGR lead to their pharmacological effects.

Similarly, Podobnic et al. combined crystallography with HDX-MS and modelling to gain insights into the structure and dynamics of the lysenin membrane pore assembly [52]. Membrane pores formed from pore-forming toxins (PFTs) are powerful molecules that, when assembled in a cell membrane, result in cell death or other detrimental effects. In their article, Podobnic et al. reveal the crystal structure of the lysenin membrane pore formed as a homononameric assembly of lysenin protomers. The arrangement of the lysenin pore assembly features a large bulbous extracellular domain, embedded into the membrane bilayer by a β-barrel which functions as a porous channel. The authors demonstrate a second utility of HDX-MS by using it as an independent method of diagnosis for the correct shape and position of the membrane pore when extracted from liposomes. The authors observe little to no exchange occurring in the transmembrane regions of the assembly, presumably due to the low solvent accessibility of these regions, while high exchange occurs in the extracellular domain. These observations were used to rationalise that the membrane pore assembly was preserved in the correct orientation. To understand the mechanism of pore formation, the authors perform MD simulations to identify the modes of flexibility of the individual lysenin protomers and parallel their findings with further HDX-MS. Flexible hinge points identified from MD readily underwent deuterium uptake, corroborating with their hypothesis that flexibility of the tongue region of each lysenin monomer contributes to the assembly pathway of the membrane pore.

**Figure 2.**
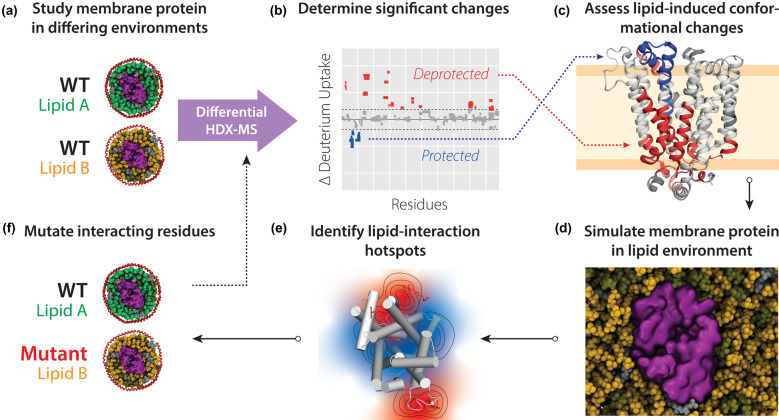
Cross-talk between HDX-MS and MD simulations allow probing of lipid-modulated conformational changes of membrane proteins. (**a**) Membrane proteins can be embedded into nanodiscs of controlled lipid compositions and subjected to differential HDX-MS. (**b** and **c**) Differential HDX-MS can reveal regions of conformational change, induced by differences in environmental lipids. (**d** and **e**) Atomistic microsecond simulations of membrane proteins in lipid environments identical with the experimental setup can be used to reveal protein–lipid interaction hotspots. (**f**) Protein residues interacting with lipids can be mutated and further tested in differential HDX-MS to test hypotheses.

Continuing on membrane proteins, a recent protocol combined HDX-MS and MD simulations to investigate the role of lipids in modulating the conformational dynamics of membrane proteins from the Major facilitator superfamily of transporters, namely XylE, LacY and GlpT [22,32] (Figure 2). HDX-MS data was first collected for various membrane proteins reconstituted in lipid nanodiscs with tuneable compositions. Explicit solvent atomistic MD simulations were carried out to determine putative sites of protein–lipid interaction. The identified sites were then tested through further HDX-MS combined with site directed mutagenesis. Corey et al. [53] integrated MD simulations with HDX-MS to monitor interactions in SecYEG and SecA interactions modulated by ATP. The authors observe that the cytoplasmic cavity of SecA widens when ATP is bound. They further performed explicit solvent atomistic simulations of the SecA-SecYEG and observe that the widening is paralleled by a reduced degree of pre-protein secondary structure compared with the external cavity. This was diminished in the ADP bound state, suggesting that ATP is used to control secondary structure formation of the pre-protein. On the same system Ahdash et al. utilised HDX-MS to study the interactions between SecA and SecYEG as a function of ATP/ADP binding [54]. Authors re-analyse an MD trajectory of the SecYE and SecA loaded with a stretch of pre-protein and analyse the size of the two pores of SecYE. Results indicated that ATP-binding leads to the constriction of the cytoplasmic side of the SecYE pore.

In another important study Nielsen et al. [25] studied the Na^+^ and dopamine-induced conformational dynamics of DAT from Drosophila (dDAT) using HDX-MS and MD simulations. The authors performed explicit solvent atomistic simulations of dDAT, in DDM detergent micelle and in 3:1:1:1 ratio bilayer of POPC, POPE, POPG and cholesterol. They found that the dynamics of dDAT in the two environments are similar, indicating that a detergent micelle *ex silico* is likely representative of the native protein.

Komives et al. [55] developed a new workflow for pairing ‘accelerated MD’ (AMD) simulations with experimental peptide-length HDX-MS data to identify the structural motions associated with transition to exchange-competent states. From the AMD simulations, the authors gathered dynamical information for motions typically observed on millisecond timescales, far beyond the timescales accessible to conventional MD. A free-energy reweighting protocol was then applied to calculate the free-energy weighted (i.e. equilibrium) population statistics for each conformer identified in the simulation. Free-energy weighted ensembles were then analysed in terms of local contacts and surface accessibility of each amide proton to classify exchange-competent and non-competent conformations. The subsequently generated exchange propensity map showed likelihood of exchange for each amide proton from the equilibrium protein dynamics on the microsecond timescale, and agreed well with observed HDX in the fast limit.

Adhikary and colleagues investigated the LeuT protein, a thermostable eubacterial neurotransmitter:sodium symporter homologue [20]. LeuT is a frequently used model system to study the conformational mechanism of mammalian neurotransmitter transport. Point mutations of LeuT are a useful tool to shift the transporter conformational ensembles between outward- and inward-facing states and have been used to explore LeuT structure with a number of techniques including X-ray crystallography, cysteine accessibility, and solution spectroscopy. To explore LeuT dynamics, the authors reconstituted wild-type and mutant transporters in lipid nanodiscs and subjected them to HDX-MS. The outward and inward-open conformational ensembles of LeuT were mimicked *in silico* through atomistic MD simulations, and residue protection factors were predicted directly from the MD structural ensembles as a function of the hydrogen bonds and local contacts around each amide hydrogen [40]. Wild-type LeuT HDX-MS at the peptide level correlated well with predicted HDX from outward-facing ensembles, while mutant HDX-MS correlated well with inward-facing ensembles. HDX-MS therefore corroborates existing evidence for LeuT conformation but also allows the interrogation of transporter dynamics with far greater structural coverage and resolution than previous methods.

Pester and co-workers studied the dynamics of amyloid-β (Aβ) peptide by combining HDX-MS, MD simulations, circular dichroism and NMR [56]. Aβ peptides produced after intramembranal cleavage of the amyloid precursor protein (C99) by γ-secretase. Cleavage is thought to occur only when C99 is a homodimer. HDX-MS performed on C99 showed that homodimerisation domain exchanges much quicker than the C-terminal cleavage site. MD simulations were performed to investigate the backbone dynamics of dimeric C99 in the presence of lipids and revealed a flexible hinge point between the dimerization and cleavage sites.

Although these successful studies demonstrate the possibility for molecular simulations to add valuable structural insights to HDX-MS experiments, quantitative interpretation of HDX-MS is often fraught with difficulty owing to the multiple sources of uncertainty (experimental, structural sampling, and predictive model) involved. To overcome these limitations, so-called ‘ensemble refinement’ techniques are frequently used to couple biophysical data to simulations in a statistically robust fashion, accounting for the uncertainties present in both the observed and predicted data [57]. Very recently, these approaches have been actively extended to HDX-MS data. Voelz et al. [58] used lengthy biased simulations and Markov state modelling to generate conformational ensembles of apo-myoglobin with a broad range of predicted HDX-MS. Using a Bayesian inference approach they then extract the most probable thermodynamical ensemble in agreement with both experimental HDX-MS and NMR chemical shift data, and identify the statistically most likely sub-populations of conformational states. In another recent study, the Forrest and Faraldo-Gómez groups developed a maximum-entropy-based procedure to reweight a mixed ensemble of structures to best fit a given set of target HDX-MS data [59]. In developing the method they show that the well-used phenomenological model for predicting HDX protection factors can discern between protein conformations with a high degree of structural fidelity, including in situations with limited experimental peptide coverage or low resolution. They exemplify the approach by re-interpreting the LeuT experimental HDX-MS data of Adhikary et al. and again ultimately identify outward- and inward-facing conformational populations of wild-type and mutant LeuT in agreement with other biophysical data. As HDX-MS experiments are more widely used to probe the functional mechanisms of biomolecules, we anticipate these methodologies will become more important to make quantitative, unbiased, and statistically robust interpretations of the observed data.

While these representative studies showcase the depth and breadth of applications and methodologies used in combining HDX-MS with MD, they are not in any way exhaustive. For more information, we suggest a number of excellent reviews [33,60–62].

## Conclusions and future outlook

We have outlined here recent advances in structural predictions from HDX-MS and molecular modelling tools. While such advances have led to novel insights and applications, in particular with respect to membrane proteins, it is worth noting that these methods remain in their infancy. Going forward, a key challenge worth noting is the discrepancy between the timescales of the experimental HDX-MS measurements and those represented by molecular simulations. While ongoing developments in microfluidics have enabled sub-second experiments, these remain well above the microsecond range achieved by atomistic level simulations. The utility of pairing together HDX-MS and molecular simulations is evident from the studies included in this review. Overall, the continued marriage between HDX-MS and molecular modelling, offers immense potential to solve current and future problems in resolving the complex and dynamic motions of biomolecules for medical and structural biology.

## Perspectives

- HDX-MS in combination with data-driven modelling can probe dynamic mechanisms underlying membrane protein structure and function
- While the merging of HDX-MS and molecular simulations remains a powerful combination, currently there are discrepancies associated with timescales and methodologies for integrating experiments with modelling.
- Continuing improvements in HDX-MS and molecular modelling, poses the immense potential to address future problems in understanding increasing large and dynamic motions of biomolecules important for human health and disease.

## Open Access

Open access for this article was enabled by the participation of King's College London in an all-inclusive Read & Publish pilot with Portland Press and the Biochemical Society under a transformative agreement with JISC.

## Abbreviations

AMD: accelerated molecular dynamics
EM: electron microscopy
PF: protection factor
PFTs: pore-forming toxins

## References

[1] Alam A., Kowal J., Broude E., Roninson I. and Locher K.P. (2019) Structural insight into substrate and inhibitor discrimination by human P-glycoprotein. Science (New York, N.Y.) 363, 753–756 10.1126/science.aav7102 PMC6800160 30765569

[2] Koshy C., Schweikhard E.S., Gartner R.M., Perez C., Yildiz O. and Ziegler C. (2013) Structural evidence for functional lipid interactions in the betaine transporter BetP. EMBO J. 32, 3096–3105 10.1038/emboj.2013.226 24141878 PMC3844952

[3] Hanson M.A., Cherezov V., Griffith M.T., Roth C.B., Jaakola V.P., Chien E.Y. et al. (2008) A specific cholesterol binding site is established by the 2.8 A structure of the human beta2-adrenergic receptor. Structure 16, 897–905 10.1016/j.str.2008.05.001 18547522 PMC2601552

[4] Kapsalis C., Wang B., El Mkami H., Pitt S.J., Schnell J.R., Smith T.K. et al. (2019) Allosteric activation of an ion channel triggered by modification of mechanosensitive nano-pockets. Nat. Commun. 10, 4619 10.1038/s41467-019-12591-x 31601809 PMC6787021

[5] Martens C., Stein R.A., Masureel M., Roth A., Mishra S., Dawaliby R. et al. (2016) Lipids modulate the conformational dynamics of a secondary multidrug transporter. Nat. Struct. Mol. Biol. 23, 744–751 10.1038/nsmb.3262 27399258 PMC5248563

[6] Pliotas C. (2017) Ion channel conformation and oligomerization assessment by site-directed spin labeling and pulsed-EPR. Methods Enzymol. 594, 203–242 10.1016/bs.mie.2017.05.013 28779841

[7] Hernandez H. and Robinson C.V. (2007) Determining the stoichiometry and interactions of macromolecular assemblies from mass spectrometry. Nat. Protoc. 2, 715–726 10.1038/nprot.2007.73 17406634

[8] Ahdash Z., Lau A.M., Byrne R.T., Lammens K., Stuetzer A., Urlaub H. et al. (2017) Mechanistic insight into the assembly of the HerA-NurA helicase-nuclease DNA end resection complex. Nucleic Acids Res 45, 12025–12038 10.1093/nar/gkx890 29149348 PMC5715905

[9] Benesch J.L. and Ruotolo B.T. (2011) Mass spectrometry: come of age for structural and dynamical biology. Curr. Opin. Struct. Biol. 21, 641–649 10.1016/j.sbi.2011.08.002 21880480 PMC3193349

[10] Barrera N.P., Isaacson S.C., Zhou M., Bavro V.N., Welch A., Schaedler T.A. et al. (2009) Mass spectrometry of membrane transporters reveals subunit stoichiometry and interactions. Nat. Methods 6, 585–587 10.1038/nmeth.1347 19578383 PMC4066579

[11] Corey R.A., Pyle E., Allen W.J., Watkins D.W., Casiraghi M., Miroux B. et al. (2018) Specific cardiolipin-SecY interactions are required for proton-motive force stimulation of protein secretion. Proc. Natl Acad. Sci. U.S.A. 115, 7967–7972 10.1073/pnas.1721536115 30012626 PMC6077702

[12] Jurneczko E., Cruickshank F., Porrini M., Clarke D.J., Campuzano I.D., Morris M. et al. (2013) Probing the conformational diversity of cancer-associated mutations in p53 with ion-mobility mass spectrometry. Angew. Chem. Int. Ed. Engl. 52, 4370–4374 10.1002/anie.201210015 23494921

[13] Konijnenberg A., Yilmaz D., Ingolfsson H.I., Dimitrova A., Marrink S.J., Li Z. et al. (2014) Global structural changes of an ion channel during its gating are followed by ion mobility mass spectrometry. Proc. Natl Acad. Sci. U.S.A. 111, 17170–5 10.1073/pnas.1413118111 25404294 PMC4260606

[14] Politis A. and Schmidt C. (2018) Structural characterisation of medically relevant protein assemblies by integrating mass spectrometry with computational modelling. J. Proteomics 175, 34–41 10.1016/j.jprot.2017.04.019 28461040

[15] Zhou M., Morgner N., Barrera N.P., Politis A., Isaacson S.C., Matak-Vinkovic D. et al. (2011) Mass spectrometry of intact V-type ATPases reveals bound lipids and the effects of nucleotide binding. Science (New York, N.Y.) 334, 380–385 10.1126/science.1210148 PMC3927129 22021858

[16] Zhou M., Politis A., Davies R., Liko I., Wu K.J., Stewart A.G. et al. (2014) Ion mobility-mass spectrometry of a rotary ATPase reveals ATP-induced reduction in conformational flexibility. Nat. Chem. 6, 208–215 10.1038/nchem.1868 24557135 PMC4067995

[17] Engen J.R. (2003) Analysis of protein complexes with hydrogen exchange and mass spectrometry. Analyst 128, 623–628 10.1039/b212800b 12866878

[18] Konermann L., Pan J. and Liu Y.H. (2011) Hydrogen exchange mass spectrometry for studying protein structure and dynamics. Chem. Soc. Rev. 40, 1224–1234 10.1039/C0CS00113A 21173980

[19] Komives E.A. (2005) Protein-protein interaction dynamics by amide H/H-2 exchange mass spectrometry. Int. J. Mass Spectrom. 240, 285–290 10.1016/j.ijms.2004.09.016

[20] Adhikary S., Deredge D.J., Nagarajan A., Forrest L.R., Wintrode P.L. and Singh S.K. (2017) Conformational dynamics of a neurotransmitter:sodium symporter in a lipid bilayer. Proc. Natl Acad. Sci. U.S.A. 114, E1786–E1795 10.1073/pnas.1613293114 28223522 PMC5347597

[21] Eisinger M.L., Dorrbaum A.R., Michel H., Padan E. and Langer J.D. (2017) Ligand-induced conformational dynamics of the *Escherichia coli* Na^+^/H^+^ antiporter NhaA revealed by hydrogen/deuterium exchange mass spectrometry. Proc. Natl Acad. Sci. U.S.A. 114, 11691–11696 10.1073/pnas.1703422114 29078272 PMC5676877

[22] Martens C., Shekhar M., Borysik A.J., Reading E., Lau A.M., Tajkorshid E. et al. (2018) Direct protein-lipid interactions shape the conformational landscape of secondary transporters. Nat. Commun. 9, 4151 10.1038/s41467-018-06704-1 30297844 PMC6175955

[23] Mistarz U.H., Brown J.M., Haselmann K.F. and Rand K.D. (2016) Probing the binding interfaces of protein complexes using gas-phase H/D exchange mass spectrometry. Structure 24, 310–318 10.1016/j.str.2015.11.013 26749447

[24] Moller I.R., Slivacka M., Nielsen A.K., Rasmussen S.G.F., Gether U., Loland C.J. et al. (2019) Conformational dynamics of the human serotonin transporter during substrate and drug binding. Nat. Commun. 10, 1687 10.1038/s41467-019-09675-z 30976000 PMC6459873

[25] Nielsen A.K., Moller I.R., Wang Y., Rasmussen S.G.F., Lindorff-Larsen K., Rand K.D. et al. (2019) Substrate-induced conformational dynamics of the dopamine transporter. Nat. Commun. 10, 2714 10.1038/s41467-019-10449-w 31221956 PMC6586795

[26] Reading E., Hall Z., Martens C., Haghighi T., Findlay H., Ahdash Z. et al. (2017) Interrogating membrane protein conformational dynamics within native lipid compositions. Angew. Chem. Int. Ed. Engl. 56, 15654–15657 10.1002/anie.201709657 29049865

[27] Masson G.R., Burke J.E., Ahn N.G., Anand G.S., Borchers C., Brier S. et al. (2019) Recommendations for performing, interpreting and reporting hydrogen deuterium exchange mass spectrometry (HDX-MS) experiments. Nat. Methods 16, 595–602 10.1038/s41592-019-0459-y 31249422 PMC6614034

[28] Chalmers M.J., Busby S.A., Pascal B.D., West G.M. and Griffin P.R. (2011) Differential hydrogen/deuterium exchange mass spectrometry analysis of protein-ligand interactions. Expert Rev. Proteomics 8, 43–59 10.1586/epr.10.109 21329427 PMC3113475

[29] Lau A.M.C., Ahdash Z., Martens C. and Politis A. (2019) Deuteros: software for rapid analysis and visualization of data from differential hydrogen deuterium exchange-mass spectrometry. Bioinformatics 35, 3171–3173 10.1093/bioinformatics/btz022 30649183 PMC6736138

[30] Hourdel V., Volant S., O'Brien D.P., Chenal A., Chamot-Rooke J., Dillies M.A. et al. (2016) MEMHDX: an interactive tool to expedite the statistical validation and visualization of large HDX-MS datasets. Bioinformatics 32, 3413–3419 10.1093/bioinformatics/btw420 27412089 PMC5181559

[31] Bouyssié D., Lesne J., Locard-Paulet M., Albigot R., Burlet-Schiltz O. and Marcoux J. (2019) HDX-Viewer: interactive 3D visualization of hydrogen-deuterium exchange data. Bioinformatics 35, 5331–5333 10.1093/bioinformatics/btz550 31287496 PMC6954641

[32] Martens C., Shekhar M., Lau A.M., Tajkorshid E. and Politis A. (2019) Integrating hydrogen-deuterium exchange mass spectrometry (HDX-MS) with molecular dynamics (MD) simulations to probe lipid-modulated conformational changes in membrane proteins. Nat. Protoc. 14, 3183–3204 10.1038/s41596-019-0219-6 31605097 PMC7058097

[33] Martens C. and Politis A. (2020) A glimpse into the molecular mechanism of integral membrane proteins through hydrogen-deuterium exchange mass spectrometry. Protein Sci. 29, 1285–1301 10.1002/pro.3853 32170968 PMC7255514

[34] Politis A., Stengel F., Hall Z., Hernandez H., Leitner A., Waltzhoeni T. et al. (2014) A mass spectrometry-based hybrid method for structural modelling of protein complexes. Nat. Methods 11, 403–406 10.1038/nmeth.2841 24509631 PMC3972104

[35] Bullock J.M.A., Sen N., Thalassinos K. and Topf M. (2018) Modeling protein complexes using restraints from crosslinking mass spectrometry. Structure 26, 1015–1024.e2 10.1016/j.str.2018.04.016 29804821 PMC6039719

[36] Hall Z., Schmidt C. and Politis A. (2016) Uncovering the early assembly mechanism for amyloidogenic beta2-microglobulin using cross-linking and native mass spectrometry. J. Biol. Chem. 291, 4626–4637 10.1074/jbc.M115.691063 26655720 PMC4813486

[37] Hansen K., Lau A.M., Giles K., McDonnell J., Sutton B.K. and Politis A. (2018) A mass spectrometry-based modelling workflow for accurate prediction of IgG antibody conformations in the gas phase. Angew. Chem. Int. Ed. Engl. 57, 17194–17199 10.1002/anie.201812018 30408305 PMC6392142

[38] Politis A., Schmidt C., Tjioe E., Sandercock A.M., Lasker K., Gordiyenko Y. et al. (2015) Topological models of heteromeric protein assemblies from mass spectrometry: application to the yeast eIF3:eIF5 complex. Chem. Biol. 22, 117–128 10.1016/j.chembiol.2014.11.010 25544043 PMC4306531

[39] Devaurs D., Papanastasiou M., Antunes D.A., Abella J.R., Ricklin D., Lambris J.D. et al. (2018) Native state of complement protein C3d analysed via hydrogen exchange and conformational sampling. Int. J. Comput. Biol. Drug Des. 11, 90–113 10.1504/IJCBDD.2018.090834 30700993 PMC6349257

[40] Best R.B. and Vendruscolo M. (2006) Structural interpretation of hydrogen exchange protection factors in proteins: characterization of the native state fluctuations of CI2. Structure 14, 97–106 10.1016/j.str.2005.09.012 16407069

[41] Vendruscolo M., Paci E., Dobson C.M. and Karplus M. (2003) Rare fluctuations of native proteins sampled by equilibrium hydrogen exchange. J. Am. Chem. Soc. 125, 15686–7 10.1021/ja036523z 14677926

[42] Zhang M.M., Beno B.R., Huang R.Y., Adhikari J., Deyanova E.G. Li J. et al. (2019) An integrated approach for determining a protein-protein binding interface in solution and an evaluation of hydrogen-deuterium exchange kinetics for adjudicating candidate docking models. Anal. Chem. 91, 15709–15717 10.1021/acs.analchem.9b03879 31710208 PMC7497726

[43] Claesen J. and Politis A. (2019) POPPet: a new method to predict the protection factor of backbone amide hydrogens. J. Am. Soc. Mass Spectrom. 30, 67–76 10.1007/s13361-018-2068-x 30338451 PMC6318252

[44] Persson F. and Halle B. (2015) How amide hydrogens exchange in native proteins. Proc. Natl Acad. Sci. U.S.A. 112, 10383–10388 10.1073/pnas.1506079112 26195754 PMC4547260

[45] Hilser V.J. and Freire E. (1996) Structure-based calculation of the equilibrium folding pathway of proteins. correlation with hydrogen exchange protection factors. J. Mol. Biol. 262, 756–772 10.1006/jmbi.1996.0550 8876652

[46] Mohammadiarani H., Shaw V.S., Neubig R.R. and Vashisth H. (2018) Interpreting hydrogen-deuterium exchange events in proteins using atomistic simulations: case studies on regulators of G-protein signaling proteins. J. Phys. Chem. B 122, 9314–9323 10.1021/acs.jpcb.8b07494 30222348 PMC6430106

[47] McAllister R.G. and Konermann L. (2015) Challenges in the interpretation of protein H/D exchange data: a molecular dynamics simulation perspective. Biochemistry 54, 2683–2692 10.1021/acs.biochem.5b00215 25860179

[48] Harrison R.A., Lu J., Carrasco M., Hunter J., Manandhar A., Gondi S. et al. (2016) Structural dynamics in Ras and related proteins upon nucleotide switching. J. Mol. Biol. 28, 4723–4735 10.1016/j.jmb.2016.10.017 PMC5661953 27751724

[49] Singh J. and Udgaonkar J.B. (2016) Unraveling the molecular mechanism of pH-induced misfolding and oligomerization of the prion protein. J. Mol. Biol. 428, 1345–1355 10.1016/j.jmb.2016.01.030 26854758

[50] Xiao Y., Shaw G.S. and Konermann L. (2017) Calcium-mediated control of S100 proteins: allosteric communication via an agitator/Signal blocking mechanism. J. Am. Chem. Soc. 139, 11460–11470 10.1021/jacs.7b04380 28758397

[51] Zhang H., Qiao A., Yang D., Yang L., Dai A., De Graaf C. et al. (2017) Structure of the full-length glucagon class B G-protein-coupled receptor. Nature 546, 259–264 10.1038/nature22363 28514451 PMC5492955

[52] Podobnik M., Savory P., Rojko N., Kisovec M., Wood N., Hambley R. et al. (2016) Crystal structure of an invertebrate cytolysin pore reveals unique properties and mechanism of assembly. Nat. Commun. 7, 11598 10.1038/ncomms11598 27176125 PMC4865846

[53] Corey R.A., Ahdash Z., Shah A., Pyle E., Allen W.J., Fessl T. et al. (2019) ATP-induced asymmetric pre-protein folding as a driver of protein translocation through the Sec machinery. eLife 8, e41803 10.7554/eLife.41803 30601115 PMC6335059

[54] Ahdash Z., Pyle E., Allen W.J., Corey R.A., Collinson I. and Politis A. (2019) HDX-MS reveals nucleotide-dependent, anti-correlated opening and closure of SecA and SecY channels of the bacterial translocon. eLife 8, e47402 10.7554/eLife.47402 31290743 PMC6639072

[55] Markwick P.R.L., Peacock R.B. and Komives E.A. (2019) Accurate prediction of amide exchange in the fast limit reveals thrombin allostery. Biophys. J. 116, 49–56 10.1016/j.bpj.2018.11.023 30558884 PMC6342732

[56] Pester O., Barrett P.J., Hornburg D., Hornburg P., Pröbstle R., Widmaier S. et al. (2013) The backbone dynamics of the amyloid precursor protein transmembrane helix provides a rationale for the sequential cleavage mechanism of γ-secretase. J. Am. Chem. Soc. 135, 1317–1329 10.1021/ja3112093 23265086 PMC3560327

[57] Orioli S., Larsen A.H., Bottaro S. and Lindorff-Larsen K. (2020) How to learn from inconsistencies: Integrating molecular simulations with experimental data. Prog. Mol. Biol. Transl. Sci. 170, 123–176 10.1016/bs.pmbts.2019.12.006 32145944

[58] Wan H., Ge Y., Razavi A. and Voelz V.A. (2020) Exchange data using Bayesian inference and multiensemble Markov state models. J. Chem. Theory Comput. 16, 1333–1348 10.1021/acs.jctc.9b01240 31917926

[59] Bradshaw R.T., Marinelli F., Faraldo-Gómez J.D. and Forrest L.R. (2020) Interpretation of HDX data by maximum-entropy reweighting of simulated structural ensembles. Biophys. J. 118, 1649–1664 10.1016/j.bpj.2020.02.005 32105651 PMC7136279

[60] Huang L., So P.K. and Yao Z.P. (2019) Protein dynamics revealed by hydrogen/deuterium exchange mass spectrometry: correlation between experiments and simulation. Rapid Commun. Mass Spectrom. 33, 83–89 10.1002/rcm.8307 30321473

[61] Collier G. and Ortiz V. (2013) Emerging computational approaches for the study of protein allostery. Arch. Biochem. Biophys. 538, 6–15 10.1016/j.abb.2013.07.025 23933229

[62] Bolla J.R., Agasid M.T., Mehmood S. and Robinson C.V. (2019) Membrane protein–lipid interactions probed using mass spectrometry. Annu. Rev. Biochem. 88, 85–111 10.1146/annurev-biochem-013118-111508 30901263

